# Widening of the Field of Abdominal Surgery from Life-Saving to Health-Restoring Operations

**Published:** 1913-02

**Authors:** 


WIDENING OF THE FIELD OF ABDOMINAL SURGERY FROM LIFE-SAVING TO HEALTH-RESTORING OPERATIONS.—Arthur E. Giles, London, *Dominion Med. Mo.*, December, 1912. We quote only the statistics of abdominal operations at the Chelsea Hosptial for Women:

| Years. | No. of Abdominal Operations. | No. of Deaths. | Percentage Mortality. |
|---|---|---|---|
| 1886—1890 .......... | 126 | 27 | 21.4 |
| 1891—1895 .......... | 206 | 35 | 17.0 |
| 1896—1900 .......... | 879 | 50 | 5.6 |
| 1901—1905 .......... | 1,493 | 63 | 4.2 |
| 1906—1910 .......... | 1,880 | 54 | 2.8 |

OPERATIONS FOR TUBAL DISEASE.

| Years. | No. of Operations. | No. of Deaths. | Percentage Mortality. |
|---|---|---|---|
| 1886—1890 ........... | 12 | 4 | 33.3 |
| 1891—1895 ........... | 22 | 3 | 13.6 |
| 1896—1900 ........... | 198 | 7 | 3.5 |
| 1901—1905 ........... | 302 | 10 | 3.3 |
| 1906—1910 ........... | 363 | 5 | 1.3 |

HYSTERECTOMY FOR FIBROIDS OF THE UTERUS.

| Years. | No. of Operations. | No. of Deaths. | Percentage Mortality. |
|---|---|---|---|
| 1886—1890 ........... | 14 | 5 | 35.7 |
| 1891—1895 ........... | 12 | 5 | 41.6 |
| 1896—1900 ........... | 150 | 16 | 10:6 |
| 1901—1905 ........... | 345 | 18 | 5.2 |
| 1906—1910 ........... | 487 | 9 | 1.8 |

